# Early repolarization and the severity of coronary artery disease: A case-control study

**DOI:** 10.22088/cjim.12.4.526

**Published:** 2021

**Authors:** Roholla Hemmati, Yousef Mohsenzadeh, Shirvan Salaminia, Fatemeh Sayehmiri

**Affiliations:** 1 Department of Cardiology, School of Medicine, Ilam University of Medical Sciences, Ilam, Iran; 2 Department of Cardiac Surgery, Yasuj University of Medical Science, Yasuj, Iran; 3 Student Research Committee, Proteomics Research Center, Shahid Beheshti University of Medical Sciences, Tehran, Iran

**Keywords:** Early repolarization, Coronary artery disease, Electrocardiogram, Angiography

## Abstract

**Background:**

Early repolarization (ER) is a common finding of the routine electrocardiogram (ECG). The ER usually considered a benign ECG finding, nevertheless a controversy. This study was conducted to investigate the relationship between early repolarization (ER) and the severity of coronary artery disease in patients with a diagnostic coronary angiography.

**Methods:**

This case-control study included ninety patients (45 patients and 45 control groups) with a diagnostic angiography and was conducted in 2015. After obtaining informed consent, patients with angiography for ER were considered as cases and those for other purposes were as controls. Data were analyzed using SPSS software Version 16. A p-value lesser than 0.05 was significant.

**Results:**

The frequency of ER was higher in men (75.6%), and there was a significant relationship between sex and ER (P=0.003). The mean age of the patients in the ER group was lower than that of non-ER patients, but not statistically significant (P=0.1). Abnormal angiography was more prevalent in patients with ER than non-ER patients (59.6% vs. 40.4%). ER morphology showed a significant correlation to abnormal angiography and also stenosis severity (P=0.035).

**Conclusion:**

ER was higher in men than in women. There was a significant correlation between the existence of ER morphology and atherosclerosis severity. Screening programs for ER detection may reduce the risk of arrhythmias and prevent related complications.

Early repolarization is common and characterizes by QRS-ST junction (J-point) elevation on standard 12-lead electrocardiography (ECG). Early repolarization (ER) is usually said as a J-point elevation of more than 0.1 mV in two adjacent leads. More commonly ER has a notch that immediately follows a positive QRS wave and before ST-segment. Another morphology is a slope at the end of QRS-wave (concealed J-wave or J-point elevation), which was considered a normal variation in QRS-T wave junction for a long time (1). Even though it is usually considered as a benign finding, the potential for arrhythmias was approved in some investigations (2, 3). The ER prevalence has different reports in the general population and varies from 5.8 to 13.1% (3). The incidence is higher in young people, male sex, athletes, and African-American descent. ER frequently presents in the absence of cardiovascular disease (4). However, studies have shown that the presence of ER in inferior leads, or the higher J-point, accompanies an increase in fatal arrhythmias or sudden cardiac death (SCD) in patients without structural cardiac disease (4).

ER prevalence upsurges (16 to 25%) in men after adolescence but drops in women (11-4%), indicating the role of sex hormones (5, 6). The prevalence of ER dramatically increases in patients with idiopathic ventricular fibrillation (IVF), with an incidence of 15-70% reported in some investigations (6, 7). The pattern of occurrence could be sporadic or inherited, though the risk increases by 2-3 times in the first-degree family members (8). 

The mechanisms for both ST elevation and ventricular fibrillation are similar in the early stages of acute myocardial ischemia and the J-wave inheritance syndrome (9). Also, in patients with short QT syndrome and cardiomyopathy, ER is more common and related to arrhythmias (10). Preceding inquiries have scrutinized the relationship between ER and coronary artery disease (CAD) and showed that patients with ER ≥0.1 mV have an increased risk of cardiac events appropriate for age and gender (11). Hence, the ER pattern potentially implies an increase in mortality due to vulnerable repolarization in susceptible clinical situations (such as short QT syndrome, Broncho dilation syndrome, or coronary artery disease) in the general population.

So far, it remains controversial whether ER is a manifestation of coronary heart disease and arrhythmias or not. Thus evaluating the association between ER and coronary artery disease could be a new perspective on the assessment and management of coronary artery disease. Risk factor control or elimination is an important step forward in reducing cardiovascular disease. Since there is no study on the association of ER and coronary artery atherosclerosis, designing a study could find if the severity of atherosclerosis is higher in ER patients than patients without ER or if there is an increased risk of CAD. Therefore, this study was designed to investigate the relationship between ER and CAD in patients with a diagnostic coronary angiography in a university hospital, Ilam University of Medical Sciences, Iran.

## Methods

This study is a case-control study and included patients with a diagnostic angiography at a second-level university hospital of Ilam University of Medical Sciences, Iran, 2015. The sample size calculated by StatCalc software and Epi-info software tools and was 90 samples (45 cases and 45 controls) with a 95% confidence and 80% power. After obtaining informed consent, those patients with angiography owed to early repolarization were considered as cases, and angiographies for other reasons except ER as the controls. Exclusion criteria were patients over 70 years of age, those who were suspected to have Brugada syndrome, and patients with either long QT syndrome or short QT syndrome. A standard 12-lead ECG was performed for each patient, and two physicians anonymously reviewed the clinical information and ECGs. Data were entered in a questionnaire and were maintained privately in agreeing to the rules of the medical ethics committee.

Normal and abnormal angiograms and the severity of abnormality were compared between two groups. The presence of ER was defined as the j-point elevation of more than 0.1 millivolts and at least in two adjacent leads, either sloped (end of the QRS) or notched (at the beginning of the ST segment) and in one or two cardiac areas. Demographic characteristics, the severity of atherosclerosis, and angiographic status were recorded in both groups. At first, descriptive statistics was used for analysis. Quantitative data presented as mean and standard deviation (mean±SD) and frequencies were stated in percentages. The difference between the two groups was calculated using chi-square, Fisher’s exact, and t-test with a variable type. Two-sided student t-test was used for the comparison of the mean values. The difference between frequency distribution was classified according to Fisher's criterion and the Spearman method was used for the correlation coefficient. Data were analyzed using SPSS Version 16. The mean values and the correlation coefficient are significant at the 95% confidence level (p<0.05).

## Results

Ninety patients with coronary angiography were enrolled in this study, which consisted of 45 patients with ER as the cases and 45 patients without ER as the controls. Most of the patients with ER were males, while in the non-ER group, women were more common. The results disclosed that the ER prevalence was higher in men and there was a significant relationship (P=0.003) between gender and ER (table. 1). The mean age was lower in the ER group than those without ER (61.55±11.15 years vs. 65.68± 12.94 years), but the difference was not statistically significant. There was no statistically significant correlation between mean age and ER (P=0.1) incidence (table. 2). Patients with ER have more frequent abnormal coronary angiography than those without ER (59.6% vs. 40.4%). Most ER patients had an abnormal angiography (75.6% vs. 24.4%), while in the non-ER group, a normal angiography was more seen (51.1% vs. 48.9%). The results showed a significant relationship (P=0.035) between ER prevalence and abnormal angiography (table. 3).

The coronary artery disease was more severe in patients with ER than those of the non-ER group. Along with the increasing severity of the coronary artery stenosis, the frequency of patients increased in the ER group but decreased in the non-ER group. At the maximum degree of coronary stenosis (>90% stenosis), the majority (66.7%) was in the ER. In the ER group, the highest prevalence (81.3%) was in the range of 70-90% coronary stenosis, but only 18.8% for the non-ER group. There was a statistically significant relationship (P=0.035) between ER prevalence and the severity of stenosis (table. 4).

**Table 1:** The prevalence of ER in two groups determined by sex

Gender(frequency)	With ER	Without ER	Total number	*P*-value	*df* *₫*	*χ2†*
	n (%)	n (%)	n (%)			
Male	34(63%)	20(37%)	54(60%)	0.003	1	9.074
Female	11(30.6%)	25(69.4%)	36(40%)			
sum	45 (50%)	45(50%)	90(100)%

^₫^degree of freedom, ^†^ Chi-Square Statistic

**Table 2:** The prevalence of ER in two groups determined by age

Variable		Number	Mean SD	*P- value*
Age	With ER	45	61.5511.15	0.1
Without ER	45	65.6812.94

**Table 3:** Prevalence of abnormal angiography in ER and non-ER groups

Angiography (frequency)	With ER	Without ER	Total	*P*-value	*df₫*	*χ2†*
	n (%)	n (%)	n (%)			
Normal	11(24.4%)	22(48.9%)	33(36.7%)	0.035	4	10.36
Abnormal	34(75.6%)	23(51.1%)	57(63.3%)			
total	45(100%)	45(100%)	90(100%)

^₫^degree of freedom, ^†^ Chi-Square Statistic

**Table 4:** ER and severity of coronary stenosis

Stenosis severity (atherosclerosis)	With ER	Without ER	Total	*P*-value	*df* ^₫^	*χ2* ^†^
	n (%)	n (%)	n (%)			
<50%	11(47.8%)	12 (52.2%)	23 (25.6%)	0.035	4	10.36
50%-70%	8(53.3%)	7 (46.7%)	15(16.7%)			
70%-90%	13(81.3%)	3(18.8%)	16(17.8%)			
>90%	2(66.7%)	1(33.3%)	3(3.3%)			
normal	11(33.3%)	22(66.7%)	33(36.7%)			
total	45(50%)	45(50%)	90(100%)

^ ₫^degree of freedom, ^†^ Chi-Square Statistic

## Discussion

Atherosclerosis is the most major etiology of cardiovascular disease. Controlling and treating atherosclerosis is the foremost aim of reducing cardiovascular disease (12, 13). The chief objective of the present study was to appraise the relationship between early repolarization (ER) and the severity of atherosclerosis. The key findings of this study revealed that abnormal angiography was significantly more prevalent in patients with ER than those without ER. Furthermore, there was a significant association between ER prevalence and the severity grading of atherosclerosis. ER prevalence was more in males and with a statistically significant relationship to gender (P=0.003).

The mean age of the ER group was lower than those without ER (61.55 vs. 65.68) but was not significantly different (P=0.1). Some studies have shown that there is a significant relationship between the presence of ER morphology and younger age (2, 14). Meanwhile, the male gender, lower age, lower systolic blood pressure, higher Sokolow-Lyon index, and lower Cornell voltage were related to ER presence independently (14). In a large prospective and population-based cohort, the highest prevalence of ER was in the lowest age group (35 to 54 years). Also, there was a correlation between the presence of ER and the male sex (15). 

The synchronicity of ER morphology and male gender in the current study is an implication for the hormonal effect. A large cohort study naming coronary artery disease risk in adults (CARDIA) with 5039 participants, revealed that the black race and male gender are factors that impact the ER prevalence (16). Sex hormones could have an important role, though the reason for this higher prevalence in the male gender has not been identified clearly (17). The function of the male gender hormones in ER morphology may be like that of Brugada syndrome (17), however androgenic anabolic steroids shortened the QT interval and therefore a shortcoming of this concept (18).

Usually, ER was considered a benign ECG finding. While the ER morphology generally coupled with side effects but does not have a significant impact on mortality rate compared to other coronary risk factors (16). A few studies with contradictory findings reviewed the relationship between ER and cardiovascular events, but no study has yet evaluated ER morphology and the severity of atherosclerosis simultaneously. A large cohort study that reported an increased risk of cardiovascular events in ER morphology did not confirm the association between J-point elevation to both SCD and coronary heart disease (CHD)-related death (19).

A follow-up study on middle-aged people from the Social Insurance Institution's Coronary Heart Disease in Finland revealed that J-point elevation more than 0.1 mV was accompanied by an increased risk of cardiac death. However, there was an increase of more than 0.2 mV add-on a more significant risk of cardiac death (3).

Lee et al. showed that ER could be associated with cardiovascular events and also that CAD patients with ER could have more cardiac complications than patients without ER (20). Other studies established the relationship between ER and the risk of cardiac death (3, 21, 22). The presence of ER morphology holds a strong relationship with the development of IVF, especially in coronary artery disease, and also the incidence of arrhythmias after myocardial infarction (MI). In a meta-analysis, the death of patients with ER morphology due to arrhythmia was higher in the people without ER (23). There is a significant correlation between ER and the incidence of life-threatening ventricular arrhythmias, especially in patients with coronary artery disease (24, 25).

Reports on Palo Alto's health care system and the study of Italian olympic athletes did not confirm a meaningful bond between ER and the undesirable cardiac events and meanwhile could not show that J-wave of ER is an awful finding (14, 26). Likewise, an analysis of the results of a study of 2,234 adults in California showed that the ER did not associate with cardiovascular mortality (27). 

Most of the studies that demonstrate the association between ER and undesirable cardiovascular outcomes are the case-control studies. In contrast, negative results are mainly in longitudinal registries (14, 26). The study population and demographic features are the other reasons for these opposing consequences. Additionally, the diagnostic criteria of ER and J-wave on the electrocardiogram cause a big difference between the study findings (19).

The pathogenesis of ER development in patients without a specific cardiac structural disease is not clear. The voltage gradient of the cardiac action potential could be the mechanism responsible for the ER appearance. This mechanism occurs due to the ion channels’ activity imbalance, which produces the end of the depolarization wave (28).

In a literature review, this is the first study that reports the relationship between ER morphology and the degree of coronary atherosclerosis. The results of this study are imperative with the emphasis on screening programs and applying preventive care in ER morphology. Atherosclerosis causes hardening of the arterial wall and a decrease in vessel elasticity and ultimately a decrease in blood flow to the body organs, including the heart and brain (12, 13)

Suh et al. denoted that ER relates to coronary artery stenosis with a high predictive value, especially with moderate risk for CHD and their study directly linked ER with CHD (29). Probably ER morphology in patients with CAD is due to irreversible damage to the heart. Earlier studies have suggested pre-infarction coronary obstruction as a possible mechanism (11). Patients with healed myocardial infarction (MI) have a high prevalence of ER (22). In the study that established the relationship between ER morphology and cardiovascular disease (CVD), half of the patients with ER morphology displayed myocardial injury. In patients without myocardial injury, no correlation was seen between ER morphology and the past cardiac events (20). Thus, the assumption is that myocardial damage could have a remarkable effect. 

Also, common pathogenesis mechanisms of the ER or CAD could be another reason. One of these is the calcium-sensitive receptor (CSR) mechanism (28). CRS is a G-Protein coupled receptor in calcium hemostasis and was expressed in various cell types, including cardiomyocytes (30). Studies have discovered that CSR in the endothelial layer of the coronary arteries activates I_K. Ca_ channel, which interferes with J-wave, so impaired function or deficient CSR expression may cause ER(31). The role of CSR in vascular calcification and atherosclerosis has also been identified (32), which may be a possible explanation of the detected relationship between ER and the presence and severity of atherosclerosis in the present study.

Cardiovascular disease is still one of the most common causes of mortality in many societies, even though many preventative measures are in practice (33). Since atherosclerosis is the core basis of cardiovascular disease, identifying a warner of atherosclerosis could be a step towards reducing cardiovascular disease. Our results showed that ER morphology could be a warning for atherosclerosis screening.

**Limitations:**

First, the sample size is small in both groups, which is a prerequisite in studies of a larger sample size. Second, we could not do a regression analysis of influencing factors such as age, sex, and history of atherosclerosis. Therefore, the relationship between ER and the severity of atherosclerosis maybe not accurate without considering these confounding factors. Third, despite this fact that the study was on patient records, some of the measurements may be misclassified.

Fourth, because the study was performed among patients with diagnostic angiography at an older age, we could not generalize the effect of ER on the youth age range. Another limitation was that the entirely epidemiologic nature of this research; so, we could not clarify the causes of the observed relationship between ER and atherosclerosis. Moreover, the current study involved multivariate models that probably affected the results.

This research unveiled that the ER is more common in men and with a significant statistical correlation between sex and ER. Since the reason for this relation is not clear, more researches are needed to determine the consequential prognosis of this relationship. Abnormal coronary angiography and the severity of atherosclerosis both were significantly higher in patients with ER than those without ER. There was a statistically significant relationship between ER prevalence and atherosclerosis severity. Since similar studies are not available about the association between ER and the severity of atherosclerosis, it is a requisite to an inquiry on patients with existing atherosclerosis and measuring these results and relevant events in the long-term view. If the association was confirmed, the presence of ER could be an indicator to perform diagnostic angiography regardless of other factors. Timely diagnosis, better treatment of atherosclerosis, and preventing complications such as arrhythmias and cardiomyopathies could be the incoming results.
